# Prognostic Value of Multifrequency Bioelectrical Impedance Analysis in Chronic Obstructive Pulmonary Disease: Systematic Review

**DOI:** 10.3390/medicina61112003

**Published:** 2025-11-08

**Authors:** Loredana-Crista Tiucă, Gina Gheorghe, Vlad Alexandru Ionescu, Ninel Iacobus Antonie, Camelia Cristina Diaconu

**Affiliations:** 1 Faculty of Medicine, University of Medicine and Pharmacy Carol Davila Bucharest, 050474 Bucharest, Romania; gheorghe_gina2000@yahoo.com (G.G.); vladalexandru.ionescu92@gmail.com (V.A.I.); ninel-iacobus.antonie@drd.umfcd.ro (N.I.A.); camelia.diaconu@umfcd.ro (C.C.D.); 2 Internal Medicine Department, Clinical Emergency Hospital of Bucharest, 105402 Bucharest, Romania; 3 Academy of Romanian Scientist, 050085 Bucharest, Romania

**Keywords:** chronic obstructive pulmonary disease, copd, prognosis, mortality, bioelectrical impedance analysis, BIA, multifrequency bioelectrical impedance analysis

## Abstract

*Background and Objectives*: Chronic obstructive pulmonary disease (COPD) is a systemic condition in which muscle wasting, malnutrition, and altered fluid balance strongly influence prognosis. While spirometry remains essential for diagnosis and staging, it often fails to reflect the heterogeneity of outcomes. Multifrequency bioelectrical impedance analysis (MF-BIA) enables the assessment of body composition and fluid distribution, offering additional prognostic information. This systematic review aimed to evaluate the prognostic significance of MF-BIA in COPD, with emphasis on outcomes such as mortality, exacerbations, and hospital admissions. *Materials and Methods*: We systematically searched PubMed, Web of Science and Scopus from inception to 29 April 2025. The earliest record retrieved was published in 1996 but was excluded during screening. Studies including COPD patients in whom MF-BIA-derived parameters were related to clinical outcomes were eligible. Risk of bias was assessed using the Newcastle–Ottawa Scale. Data on design, population, methodology, and endpoints were extracted and narratively synthesized due to heterogeneity. The review protocol was not registered. *Results*: Eight studies were included. Phase angle (PhA) consistently showed prognostic value, being inversely related to mortality and rehospitalizations. Fat-free mass index (FFMI) was integrated into multidimensional models, but its independent role was inconsistent. Parameters describing fluid distribution, such as Extracellular Water/Total Body Water ratio, also appeared relevant, though interpretation was often limited by the absence of consistent consideration of underlying cardiac disease. *Conclusions*: MF-BIA provides useful prognostic insights in COPD patients, particularly through PhA. It may refine risk stratification beyond spirometry, yet further prospective studies with standardized methods are needed to confirm its independent value. Heterogeneity of methods and small sample sizes remain important limitations.

## 1. Introduction

### 1.1. Chronic Obstructive Pulmonary Disease: Systemic Involvement and Prognostic Complexity

Chronic obstructive pulmonary disease (COPD) affects approximately 10.3% of the adult population over the age of 40 years worldwide and is currently the third leading cause of death, accounting for an estimated 3.2 million deaths annually [1]. The disease burden is projected to increase further because of ongoing exposure to risk factors and population aging. Comorbidities are common in COPD, and heart failure is among the most clinically significant. Epidemiological studies report that 20–30% of patients with COPD have coexisting heart failure, a combination that is associated with higher mortality rates, more frequent hospitalizations, and increased healthcare burden compared to either condition alone [2,3].

Beyond its pulmonary manifestations, COPD is increasingly recognized as a systemic disease, with extrapulmonary features such as skeletal muscle dysfunction, malnutrition, and fluid imbalance contributing significantly to disease burden and prognosis [4,5].

COPD is characterized not only by chronic local airway inflammation but also by a persistent systemic inflammatory response, which contributes to its extrapulmonary manifestations and systemic complications. This inflammatory state affects skeletal muscle metabolism, nutritional status, and immune function, creating a complex interplay between pulmonary and systemic disease components [1].

Muscle fatigue and wasting in COPD arise through multifactorial mechanisms. On one hand, deconditioning and reduced physical activity, driven by dyspnea and ventilatory limitation, and on the other hand, inflammatory-mediated catabolism, promoted by cytokines such as tumor necrosis factor-α (TNF-α) and interleukin-6 (IL-6), enhance protein degradation and mitochondrial dysfunction within muscle fibers. These processes jointly result in the loss of skeletal muscle mass and fat-free mass (FFM), which in turn worsens respiratory mechanics, exercise tolerance, and overall prognosis [4].

The ECLIPSE study identified six systemic inflammatory biomarkers—white blood cell count, fibrinogen, C-reactive protein (CRP), IL-6, interleukin-8 (IL-8), and TNF-α—that are associated with exacerbation frequency and mortality in COPD [6]. More recently, composite indices reflecting systemic inflammation, such as the neutrophil-to-lymphocyte ratio (NLR), CRP-to-albumin ratio (CAR), and serum amyloid A (SAA), have been shown to correlate with the severity of systemic inflammation and to predict acute exacerbations of COPD and respiratory failure. The combined assessment of NLR, CAR, and SAA offers a comprehensive reflection of inflammatory status and holds predictive value for acute exacerbations with respiratory failure in patients with COPD [7].

Chronic inflammation in COPD is also linked to immune dysregulation, including altered lymphocyte subsets, reduced phagocytic activity, and impaired mucosal defense, which collectively increase susceptibility to infection and prolong exacerbations [1].

Among novel biomarkers, growth differentiation factor-15 (GDF-15) has emerged as a marker of muscle stress, inactivity, and metabolic dysregulation. Elevated GDF-15 levels are associated with a sedentary lifestyle, reduced exercise capacity, and muscle weakness in COPD, reflecting the interaction between systemic inflammation and skeletal muscle dysfunction. These alterations often coexist with cognitive impairment, and their overlap is now recognized as motoric cognitive risk (MCR) syndrome, a condition defined by the simultaneous presence of slow gait and cognitive decline, linked to adverse health outcomes and increased mortality. Although GDF-15 is not disease-specific, its elevation may serve as a screening biomarker of metabolic frailty and MCR, providing additional insight into the multidimensional vulnerability of COPD patients [8].

Collectively, these findings support the concept that systemic inflammation, immune dysfunction, and muscle wasting are interdependent processes that drive both disease progression and exacerbation risk in COPD, highlighting the importance of integrative approaches for early detection and prognostic assessment.

Alterations in body composition, particularly reductions in FFM, have been consistently associated with poor clinical outcomes, including increased mortality, frequent exacerbations, and higher rates of hospital readmission [9].

The available evidence regarding the association between bioimpedance-derived parameters and systemic inflammatory markers remains inconsistent in COPD. Some studies support a link between systemic inflammation and body composition alterations, although no causal relationship has been established [10]. While previous reports associated cachexia with elevated TNF-α levels, other investigations found no evidence that TNF-α-mediated inflammation drives muscle loss [11]. These discrepancies highlight the need for further studies to clarify the complex interplay between inflammation, body composition, and metabolic status in COPD.

COPD frequently coexists with chronic conditions that substantially influence prognosis [1]. Some comorbidities develop de novo due to shared risk factors such as smoking, aging, or systemic inflammation, while others arise secondarily through mechanisms directly linked to COPD pathophysiology [1]. These overlapping disorders, particularly cardiac, renal, hepatic, and metabolic diseases, can independently modify body composition and fluid balance.

Heart failure, chronic kidney disease, liver disease, and diabetes mellitus can alter multifrequency bioelectrical impedance analysis (MF-BIA) measurements through distinct pathophysiological pathways. Heart failure increases the extracellular water/total body water (ECW/TBW) ratio as a result of fluid redistribution [12]. Chronic kidney disease impairs extracellular volume regulation and ionic conductivity. Liver dysfunction may cause hypoalbuminemia and third-space fluid shifts, while diabetes mellitus contributes to inflammation-driven muscle atrophy and sarcopenic obesity [13].

Changes in water distribution are particularly relevant in multimorbid COPD, especially when heart failure is present, a combination that is frequent and markedly worsens prognosis [1,2]. An elevated ECW/TBW ratio reflects extracellular volume expansion, which may indicate subclinical or overt congestion in patients with heart failure or renal dysfunction. Monitoring ECW/TBW dynamically could therefore serve as an early marker of latent fluid overload, prompting timely medical assessment and targeted diagnostic investigations before decompensation becomes clinically apparent. Such an approach may positively influence COPD outcomes by enabling earlier intervention and preventing severe heart failure exacerbations, which are known to increase mortality in COPD [2,3].

While spirometry, particularly forced expiratory volume in one second (FEV_1_), remains the cornerstone of COPD diagnosis and staging, it may fail to capture systemic complexity and prognostic variability. Moreover, spirometry performed in patients with congestion can display an obstructive pattern, potentially leading to diagnostic misclassification or inaccurate disease staging [1,14].

These interactions highlight the importance of a contextual and stratified interpretation of bioimpedance data. Future studies should aim to define disease-specific reference ranges and perform stratified analyses according to comorbidity burden and severity, in order to better establish the prognostic relevance of MF-BIA parameters in COPD. Understanding these comorbidity-related variations is essential for accurately interpreting MF-BIA-derived parameters and for integrating them into multidimensional prognostic models in COPD.

### 1.2. Bioelectrical Impedance Analysis: Principles, Clinical Relevance and Diagnostic Potential

Bioelectrical impedance analysis (BIA) is a non-invasive and accessible method for evaluating body composition based on the opposition of body tissues to alternating electrical currents. Single-frequency BIA (SF-BIA), typically using a 50 kHz current, provides estimates of total body water (TBW) but relies on assumptions of homogeneous tissue conductivity, which may be compromised in diseases like COPD [15].

MF-BIA applies currents across a spectrum of frequencies (e.g., from 5 to 1000 kHz), enabling a more accurate differentiation between intracellular water (ICW) and extracellular water (ECW). Unlike SF-BIA, which primarily estimates TBW without distinguishing between compartments, MF-BIA provides a more detailed assessment of fluid distribution, offering additional insight into cellular integrity and hydration status [1,16].

The technique measures the impedance of body tissues (their opposition to an alternating electrical current) across multiple frequencies. At low frequencies, the current flows mainly through the extracellular space, as cell membranes act as capacitors. At higher frequencies, it can also pass through the cells, thus reflecting both extracellular and intracellular compartments. Mathematical modeling (commonly the Cole–Cole model) uses these frequency-dependent measurements to estimate ECW and ICW, from which TBW is derived (TBW = ECW + ICW). FFM is calculated from TBW assuming a constant hydration of lean tissue, while fat mass (FM) is obtained by subtracting FFM from TBW [15].

In addition, MF-BIA allows the determination of the phase angle (PhA), calculated as the arctangent of reactance over resistance (PhA = arctan[Xc/R]) and usually reported at 50 kHz. This parameter reflects the capacitive properties of cell membranes and serves as an indicator of cellular integrity and body cell mass [15].

Despite their accessibility and clinical appeal, BIA measurements are not directly interchangeable across devices, even when operating at similar frequencies. Differences in hardware design, electrode composition, signal processing, and population-specific prediction equations contribute to variability among analyzers. In addition, patient-related factors, such as hydration status, body temperature, and recent physical activity, can further affect measurement accuracy and reproducibility. Therefore, additional validation studies are needed to standardize BIA parameters and confirm their reliability across diverse clinical settings and patient phenotypes.

When SF-BIA and MF-BIA methods are compared, Yalin et al. (2017) reported significant differences in impedance-derived parameters between the two technologies in dialysis patients, with the best inter-device agreement observed for ECW [17]. In another study on postmenopausal women, both SF-BIA and MF-BIA deviated from Dual-energy X-ray Absorptiometry (DXA) reference measurements, underestimating FM and overestimating FFM, yet the multifrequency analyzer demonstrated better overall accuracy than the single-frequency device [18]. Furthermore, Bosy-Westphal et al. (2013) highlighted the advantages of using multiple frequencies and segmental analysis (1–1000 kHz) for improving the estimation of FFM, TBW, and ECW in healthy, euvolemic adults, although extrapolation of these results to patients with chronic diseases should be performed cautiously [19]. Shafer et al. (2009) reported that MF-BIA tends to overestimate the percentage of body fat (PBF%) compared with DXA, particularly in individuals with obesity or altered hydration [20,21].

Nevertheless, the ability of MF-BIA to differentiate ECW and ICW compartments remains a major advantage, providing clinically relevant insights into fluid distribution, inflammation, and cellular integrity, features often disrupted in patients with chronic respiratory disease.

Overall, MF-BIA offers a promising compromise between accuracy and practicality, especially considering that reference methods such as DXA, deuterium dilution, and air-displacement plethysmography, although more precise, are expensive, time-consuming, and not feasible for routine use in clinical practice [22].

The differential diagnosis between COPD exacerbations and episodes of heart failure decompensations, particularly heart failure with preserved ejection fraction (HFpEF), is frequently challenging due to overlapping clinical symptoms such as dyspnea, fatigue, and reduced exercise tolerance. Misclassification is common: episodes of decompensated heart failure may be mistakenly attributed to COPD exacerbations, resulting in delays or errors in management [4]. Paraclinical investigations also have limitations. Spirometry performed in a hypervolemic state may exhibit obstructive changes, potentially leading to a false diagnosis or misclassification of COPD severity. Likewise, N-terminal pro-B-type natriuretic peptide (NT-proBNP) levels can be elevated in COPD patients with pulmonary hypertension, which may reduce the diagnostic specificity of natriuretic peptides and necessitate higher interpretive thresholds [2].

Given these diagnostic and prognostic challenges, MF-BIA may offer valuable adjunctive data. BIA may serve as a valuable tool for assessing disease severity and prognostic risk in COPD patients by providing objective data on fluid distribution and cellular integrity. In addition, it may aid in distinguishing between cardiac and pulmonary contributors to clinical decompensation, thereby supporting more accurate phenotypic stratification in complex cases [23,24].

### 1.3. Rationale: Prognostic Value of MF-BIA in COPD

Parameters derived from MF-BIA, particularly PhA and fat-free mass index (FFMI), have emerged as valuable prognostic markers in chronic conditions, including COPD. PhA reflects cell membrane integrity and function and has shown consistent inverse associations with morbidity, mortality, and hospital readmissions in chronic respiratory patients [23,25].

Compared to SF-BIA, MF-BIA applies currents across a broad spectrum of frequencies, allowing for separate estimation of intracellular and extracellular water compartments. This multi-compartmental approach offers a theoretically improved sensitivity for detecting subtle shifts in hydration status, cellular membrane integrity, and tissue composition. Overall, MF-BIA may provide a more comprehensive characterization of body composition, although evidence confirming its clinical or prognostic superiority remains limited [15].

Recently, De Benedetto et al. (2023) [26] provided a comprehensive review on the application of BIA in respiratory diseases, emphasizing the presence of malnutrition and the clinical significance of PhA assessment in patients with respiratory disorders, including COPD. Accumulating clinical evidence indicates that lower PhA values are associated with impaired muscle strength, reduced exercise capacity, and increased all-cause mortality. However, other parameters measurable through MF-BIA, such as the differentiation of intracellular and extracellular water compartments or indices reflecting fluid distribution, remain insufficiently characterized in COPD, and their prognostic relevance is still poorly defined [26].

Although MF-BIA has demonstrated higher agreement with reference methods such as DXA and deuterium dilution in populations with stable or euvolemic hydration [21], robust evidence supporting its accuracy in patients with respiratory diseases is still lacking. However, reference techniques are expensive, time-consuming, and impractical for routine use, particularly in multimorbid or frail populations. Therefore, MF-BIA represents a promising and feasible alternative for assessing body composition and fluid distribution in COPD, with potential applications in both clinical monitoring and prognostic evaluation.

### 1.4. Objectives

This systematic review aims to evaluate the prognostic utility of MF-BIA in adult patients with COPD. Specifically, it seeks to determine whether MF-BIA-derived parameters are independently associated with clinically relevant outcomes such as mortality, exacerbation rates, hospital admissions, or disease progression.

## 2. Materials and Methods

### 2.1. Protocol and Registration

This systematic review was conducted in accordance with the Preferred Reporting Items for Systematic Reviews and Meta-Analyses (PRISMA 2020) guidelines. A detailed protocol was developed prior to data collection and analysis, outlining eligibility criteria, search strategy, and methods for data extraction and synthesis. Although the protocol was not registered in PROSPERO, the methodology strictly adhered to PRISMA 2020 recommendations. All methodological decisions were predefined to ensure transparency, consistency, and reproducibility.

### 2.2. Eligibility Criteria

This systematic review aimed to evaluate the prognostic value of MF-BIA in patients with COPD. To ensure methodological consistency and clarity, studies were selected based on predefined eligibility criteria structured according to the PICOS framework:

- Population: Adult patients (≥18 years) diagnosed with COPD, based on accepted international guidelines (e.g., GOLD). Studies with mixed populations were included only if COPD-specific results were reported separately.
- Intervention: Use of MF-BIA to assess body composition. Studies using BIA without explicit frequency specification were eligible only if BIA-derived parameters were clearly central to the prognostic model. Studies using SF-BIA were excluded.
- Comparators: Not required for eligibility. Both single-arm and comparative studies were included.
- Outcomes: Studies were required to report prognostic endpoints associated with BIA parameters, including all-cause mortality, survival, frequency of exacerbations, or hospital readmissions.
- Study design: Eligible studies included observational (prospective or retrospective cohort, case–control) and interventional studies. Reviews, meta-analyses, editorials, opinion pieces, conference abstracts without full text, and studies with insufficient methodological quality were excluded.

Other criteria:

- Language: No language restrictions were applied; full texts were evaluated directly or via assisted translation.
- Publication date: No restrictions by year of publication; studies indexed up to 29 April 2025 were eligible.
- Citation tracking: Backward and forward citation tracking was not applied.

### 2.3. Information Sources

A comprehensive literature search was conducted to identify relevant studies investigating the prognostic value of BIA in patients with COPD. The search was performed across multiple electronic databases, each queried using tailored search strategies adapted to their specific syntax and indexing structures. The following databases were systematically searched: PubMed, Scopus, and Web of Science (Core Collection).

The final search was conducted on 29 April 2025, covering the period from database inception to that date; the earliest record retrieved was published in 1996. No language or publication date restrictions were applied, provided that full-text access was available.

### 2.4. Search Strategy

The search strategy combined Medical Subject Headings (MeSH), free-text keywords, and Boolean operators to maximize the retrieval of relevant studies. The terms used reflected three key domains:

- Chronic obstructive pulmonary disease: “COPD”, “chronic obstructive pulmonary disease”.
- Bioelectrical impedance analysis: “bioimpedance”, “bioelectrical impedance”, “BIA”, “multifrequency bioimpedance”, “multi-frequency bioelectrical impedance”.
- Prognostic relevance: “prognosis”, “prognostic”, “outcome”, “mortality”, “survival”.

The Boolean operators AND and OR were used to construct logical combinations of terms, ensuring a sensitive and inclusive search strategy.

The complete search strings used for PubMed, Scopus, and Web of Science are provided in Appendix A.1 for transparency and reproducibility.

### 2.5. Selection Process

All references retrieved from the database searches were imported into Zotero (version 7.0.15; Corporation for Digital Scholarship, Vienna, VA, USA) for reference management. Duplicate records were identified and removed using a combination of automated detection and manual inspection. The remaining records were screened in two phases: initial screening of titles and abstracts, followed by full-text review of potentially eligible studies.

Screening and study selection were conducted by a single reviewer (LCT), based on the predefined inclusion and exclusion criteria outlined in Section 2.2. Although independent duplicate screening was not performed due to resource limitations, eligibility decisions were made systematically and documented in detail to ensure consistency and reproducibility.

**Figure 1:**
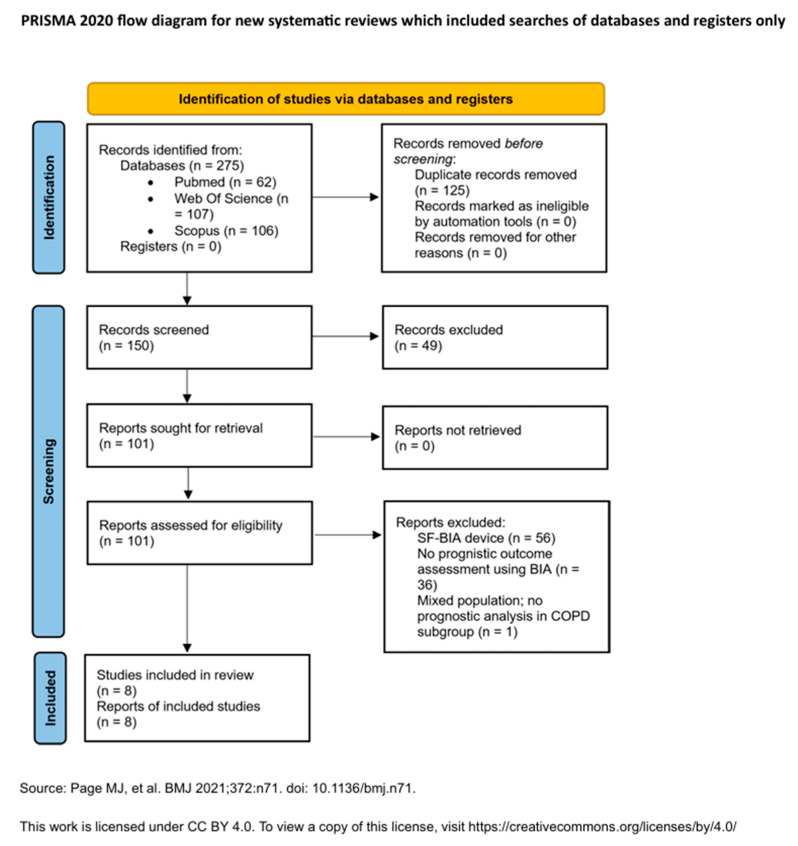
PRISMA 2020 Flow Diagram: Visual representation of the study selection process. Adapted from Page et al. [27]. This work is licensed under a CC BY 4.0 license.

The selection process and the number of records at each stage (identified, screened, excluded, and included) will be documented in a PRISMA 2020 flow diagram (Figure 1). 

### 2.6. Data Collection Process

Data from included studies were extracted by LCT using a structured extraction form developed for this review and piloted for consistency and clarity. Bibliographic management was performed in Zotero (version 7.0.15; Corporation for Digital Scholarship, Vienna, VA, USA), and extracted data were recorded in Microsoft Excel ( for synthesis and quality control.

### 2.7. Data Items

For each study, the following variables were extracted:

- Study identification: first author, year of publication and country.
- Study characteristics: study design, sample size and follow-up duration.
- Population details: baseline characteristics and relevant cardiac comorbidities, where available.
- BIA-related information: device used and BIA parameters.
- Prognostic outcomes: mortality, exacerbation rates, hospitalizations, or composite endpoints. All reported results that were compatible with these outcome domains were sought, regardless of time point or statistical model.
- Statistical analysis: effect measures, covariates used in multivariate models, confidence intervals.
- Key findings: main conclusions regarding the prognostic relevance of MF-BIA parameters.

When information was missing or unclear, it was recorded as “not reported” and no assumptions were made beyond the data explicitly provided in the original study.

In addition to the data collected, no information regarding study funding or conflicts of interest was systematically extracted, as this was not prespecified in the review protocol.

The final version of the data extraction form is presented in Appendix A.2.

### 2.8. Risk of Bias Assessment

The risk of bias was assessed using the Newcastle–Ottawa Scale (NOS), which evaluates selection, comparability, and outcome/exposure assessment. The reviewer applied predefined scoring criteria, classifying studies as having low, moderate, or high risk of bias. Although duplicate independent scoring was not performed, efforts were made to apply the criteria consistently and transparently. Full NOS scores are presented in Appendix A.3.

### 2.9. Effect Measures

The primary effect measures extracted from each study included hazard ratios (HRs), odds ratios (ORs), relative risks (RRs), correlation coefficients, and mean differences, depending on the outcome type and statistical model used.

- For time-to-event outcomes (e.g., mortality, survival), hazard ratios were preferred when available.
- For dichotomous outcomes, odds ratios or relative risks were recorded.
- For continuous outcomes, mean differences or standardized mean differences were noted.

When reported, 95% confidence intervals and *p*-values were extracted to evaluate statistical significance and estimate precision. No recalculation or transformation of effect measures was undertaken during data extraction.

### 2.10. Synthesis Methods

Studies were eligible for synthesis if they evaluated BIA parameters in relation to prognostic outcomes, specifically mortality, hospitalizations, or exacerbations. Data were extracted as reported in the original publications, without transformation.

Findings from individual studies were summarized descriptively in tabular form (see Appendix A.4), including study design, BIA methodology, effect estimates, and key results.

Due to substantial expected heterogeneity in study design, patient populations, BIA parameters, and outcome definitions, a quantitative synthesis (meta-analysis) was not considered appropriate. Instead, a structured narrative synthesis was conducted, organized according to the type of prognostic outcome assessed and the specific MF-BIA parameters evaluated (e.g., PhA, FFM, ECW/ICW ratio).

No formal exploration of heterogeneity, subgroup analyses, or sensitivity analyses were performed. Observed differences between studies were described qualitatively in the Section 3.

### 2.11. Reporting Bias Assessment

As this review did not include a meta-analysis, formal assessment of publication bias (e.g., via funnel plots) was not applicable. However, potential selective reporting within individual studies was considered during data extraction and risk of bias assessment. Suspected cases of incomplete outcome reporting or unclear descriptions of prognostic variables were noted and are reflected in the judgments presented in Section 2.8 and Appendix A.3.

### 2.12. Certainty Assessment

A formal GRADE assessment was not performed, as no meta-analysis was conducted and the included studies exhibited substantial clinical and methodological heterogeneity. Instead, the overall certainty of the evidence was evaluated qualitatively, based on key domains including risk of bias, consistency of findings, directness of evidence, and precision of reported estimates.

Studies with low risk of bias and consistent associations between MF-BIA parameters and clinical outcomes were interpreted as contributing higher certainty. Conversely, evidence from studies with methodological limitations, small sample sizes, or selective reporting was considered less reliable.

A narrative summary of the overall certainty of evidence is presented in the Section 4, along with implications for future research.

### 2.13. Use of Generative Artificial Intelligence

Generative AI (ChatGPT, GPT-5 model; OpenAI, San Francisco, CA, USA) was used to assist in language editing, text refinement, and improvement of readability. The authors critically reviewed, revised, and verified all content to ensure accuracy and scientific integrity.

## 3. Results

### 3.1. Study Selection

The initial search yielded a total of 275 records across all databases. After removing 125 duplicates using Zotero (version 7.0.15; Corporation for Digital Scholarship, Vienna, VA, USA) and manual inspection, 150 unique records remained for screening. Based on titles and abstracts, 49 studies were excluded for irrelevance. A total of 101 full-text articles were then assessed for eligibility, of which 8 met the inclusion criteria and were included in the final synthesis.

Study selection was conducted by a single reviewer (LCT), based on the predefined eligibility criteria. Reasons for exclusion at the full-text screening stage were documented and are summarized in the PRISMA 2020 flow diagram (Figure 1). A detailed list of excluded studies with reasons for exclusion is provided in Appendix A.5.

### 3.2. Study Characteristics

A total of 8 studies were included in this systematic review, published between 2015 and 2024, and conducted across diverse geographical regions, including Asia (Japan [28], China [29], South Korea [30]), Europe (Greece [31], Italy [32], United Kingdom [33]), and North America (Mexico [34]). The majority were prospective cohort studies (*n* = 5) [28,31,32,33,34], with two retrospective cohorts [30,35] and one cross-sectional study with prognostic analysis [29].

Sample sizes ranged from 76 [31] to 502 patients [33], with populations predominantly consisting of older adults. Comorbidities were reported in 4 out of 8 studies, but only 3 provided detailed information [28,30,34]. Follow-up durations ranged from 90 days [33] to 7 years [34].

BIA was performed using MF analyzers in 6 studies [28,29,31,32,33,34]. Two studies did not report device specifications but were retained as they provided relevant prognostic data [30,35]. The most used device was the InBody S10 (*n* = 3) [28,29,31], followed by the BodyStat QuadScan 4000 (*n* = 2) [33,34] and Human IM-Touch (*n* = 1) [32].

The most commonly reported BIA parameters included:

- FFM and FFMI (7 out of 8 studies) [29,30,31,32,33,34,35].
- PhA (6 studies, with segmental PhA used in selected cases) [28,29,31,32,33,34].
- Impedance ratio (two studies) [32,34].
- Skeletal muscle index (SMI) [29,30] and fat mass index (FMI) [29,30].
- ECW/TBW (1 study) [29].

Clinical outcomes varied and included:

- All-cause mortality (*n* = 5 studies) [30,32,33,34,35].
- Acute COPD exacerbations *(n* = 4 studies) [28,29,30,31].
- Hospitalizations, emergency visits, and pneumonia incidence (in multicenter or longitudinal studies).

Among the eight studies included in this review, two used the InBody S10 analyzer (InBody, Tokyo, Japan), where PhA was calculated using the standard equation PhA (°) = arctan (Xc/R) × (180/π) at a frequency of 50 kHz. One additional study employing the same device obtained PhA values at five frequencies (5–250 kHz) for both dominant and non-dominant body sides. Two studies used the Bodystat QuadScan 4000 (Bodystat Ltd., Isle of Man, UK), in which PhA was calculated using the same equation at a reference frequency of 50 kHz through the manufacturer’s software. One study applied the Human IM-Touch analyzer (DS Medica Srl, Milan, Italy), where PhA was measured at five frequencies and expressed in degrees at 50 kHz. In the remaining two studies, the BIA device was not specified and PhA was neither calculated nor reported. All devices were phase-sensitive analyzers capable of directly measuring resistance and reactance, ensuring that the reported PhA values represented true bioelectrical properties rather than model-derived estimates.

Several studies used Cox regression models, while others employed multivariate logistic regression or cluster analysis to assess the prognostic relevance of BIA-derived parameters.

### 3.3. Risk of Bias in Studies

The methodological quality of included studies, assessed via the NOS, ranged from moderate to high. Five studies were judged to have a low level of bias, while three were rated as moderate risk of bias. Most studies had clearly defined populations and outcomes, but differences in BIA methodology, cut-off definitions, and covariate adjustment may have introduced heterogeneity. These limitations underscore the need for standardization in future research (see Appendix A.3).

### 3.4. Results of Individual Studies

Table 1 provides a concise overview of the main conclusions from the eight included studies, while detailed effect estimates, confidence intervals, and model specifications are presented in Appendix A.4.

Several studies identified PhA as a significant predictor of mortality or exacerbations [28,32], particularly when measured segmentally [28]. Others highlighted the role of FFM and FFMI, although not all associations remained significant after adjustment for prior exacerbations [31]. One study applied cluster analysis based on FFMI to outperform traditional prognostic tools [35], and another reported that PhA was a stronger predictor than FFMI [33].

While most studies employed multivariate regression models, the degree of adjustment and outcomes measured varied, limiting cross-study comparability.

### 3.5. Synthesis of Results

#### 3.5.1. Mortality

Four of the included studies evaluated all-cause mortality as a primary prognostic outcome, with follow-up periods ranging from one to seven years. Across these studies, PhA emerged as the most consistently reported MF-BIA parameter associated with mortality risk. PhA was first reported as a possible prognostic factor in 2015 by Maddocks et al. [33].

Gómez-Martínez et al. (2023) conducted a 7-year prospective cohort study and found that lower PhA was significantly associated with increased mortality, both when analyzed as a continuous variable (HR = 0.59, 95% CI: 0.37–0.94, *p* = 0.026) and when dichotomized at the 50th percentile (HR = 3.47, 95% CI: 1.45–8.29, *p* = 0.005) [34]. Sarcopenia was also independently associated with mortality (HR = 2.10, 95% CI: 1.02–4.33), whereas impedance ratio did not retain statistical significance in multivariate analysis [34].

De Blasio et al. (2019) found that impedance ratio and PhA were independent predictors of 1-year mortality in a COPD population hospitalized for pulmonary rehabilitation, even after adjusting for age, comorbidities, and FEV_1_ [32].

Rodrigues et al. sought to identify clusters of patients with COPD that could predict two-year mortality, and they incorporated FFMI as one of the parameters within these clusters [35]. However, the prognostic value of FFMI remains uncertain, as other studies have not demonstrated a consistent association between this index and either mortality risk or disease severity [31,33].

Taken together, these findings support the role of PhA as prognostic BIA-derived marker for mortality in COPD patients.

#### 3.5.2. Exacerbations and Hospitalizations

The relationship between MF-BIA-derived parameters and the risk of severe exacerbations or hospital admissions in COPD patients was also of interest among the studies included. PhA was one of the most commonly evaluated parameters.

Kobayashi et al. (2024) [28] conducted a prospective cohort study with a 3-year follow-up and found that lower segmental PhA values in the lower limbs (right leg and left leg) and left arm were significantly associated with a higher risk of severe exacerbations requiring hospitalization or emergency department visits. Adjusted hazard ratios ranged from 2.61 to 3.50 depending on segment, and the highest predictive performance was observed for PhA of the right leg (AUC = 0.73, cut-off: 4.5°) [28].

Xie et al. (2024) [29], in a cross-sectional study with logistic regression-based prognostic stratification, identified low PhA and high ECW/TBW ratio as significant predictors of the frequent exacerbator phenotype. Specifically, PhA < 4.85° (AUC = 0.753) and ECW/TBW > 0.393 (AUC = 0.744) were associated with higher odds of frequent exacerbations, with adjusted ORs of 0.396 and 1.086, respectively [29]. However, this study did not address the possibility of coexisting heart disease, leaving it unclear whether the higher ECW/TBW ratio reflected changes related to COPD itself or to an associated cardiac decompensation. This underscores the difficulty in disentangling fluid imbalance due to COPD versus concomitant cardiac dysfunction.

#### 3.5.3. Functional or Composite Outcomes

Rodrigues et al. (2018) [35] performed a cluster analysis in patients with stable COPD and identified a high-risk phenotype associated with two-year mortality and exacerbations. This phenotype was defined by established predictors such as FEV_1_ and mMRC but also incorporated FFMI, a parameter derived from bioimpedance analysis. By including FFMI within a multidimensional risk model, the study underscored the potential role of BIA-derived measures in refining prognostic stratification beyond traditional spirometric and clinical indices [35].

Although individual prognostic measures were not isolated, the integration of BIA parameters into a multidimensional risk stratification model underscores their potential utility in evaluating COPD patients and could contribute to composite risk models.

### 3.6. Reporting Biases

Formal assessment of reporting bias, such as funnel plot analysis, was not feasible due to the limited number of included studies and the lack of meta-analytic pooling.

However, selective reporting cannot be ruled out. Several studies reported only statistically significant associations or did not fully disclose multivariate model adjustments. Additionally, two studies did not provide detailed information regarding BIA device specifications or measurement protocols, which may impact reproducibility.

Publication bias is also possible, given that all included studies reported at least one positive association between BIA parameters and clinical outcomes. The absence of unpublished or negative studies may overestimate the perceived prognostic utility of BIA in COPD.

### 3.7. Certainty of Evidence

The overall certainty of evidence regarding the prognostic value of BIA in COPD is moderate, supported by prospective cohort data and consistent associations of PhA and FFMI with clinical outcomes such as mortality and exacerbations.

The strengths of the current evidence include:

- Use of validated clinical endpoints (e.g., mortality, exacerbations).
- Consistent findings for PhA and FFMI across multiple studies.
- Application of multivariate statistical models in most analyses.

The limitations reducing certainty and generalizability are:

- Methodological heterogeneity.
- Incomplete adjustment for confounders such as comorbidities.
- Small sample sizes and absence of standardized outcome definitions in several studies.

These limitations highlight the need for future well-powered, prospective studies employing standardized BIA protocols and harmonized definitions of clinical endpoints.

## 4. Discussion

### 4.1. Main Findings

This systematic review synthesized evidence from eight observational studies evaluating the prognostic role of MF-BIA in patients with COPD. The results demonstrate that several BIA-derived parameters, most notably PhA, FFMI, and impedance ratio, are associated with meaningful clinical outcomes such as acute exacerbations and all-cause mortality. These parameters can be measured noninvasively, repeatedly, and safely, offering a promising avenue for dynamic risk monitoring in clinical practice.

Segmental PhA, particularly in the lower limbs, was independently predictive of severe exacerbations in male COPD patients [28], while TSMI predicted acute exacerbation risk in males [30]. Regarding mortality, lower PhA consistently emerged as a robust predictor across studies [32,33,34]. A cluster-based approach incorporating FFMI and other functional markers also effectively stratified mortality risk, although FFMI was not predictive on its own in most regression models [35]. One study reported that associations between FFMI or FFM and exacerbation risk did not remain significant after adjusting for previous exacerbation history [31].

Altogether, these findings support the hypothesis that BIA-derived markers reflect systemic alterations in body composition and cellular function that go beyond pulmonary mechanics and may be useful for guiding risk stratification and treatment tailoring in COPD.

Existing literature has consistently emphasized the prognostic implications of muscle mass loss and nutritional depletion in COPD. BIA represents an evolution of this concept by providing objective, reproducible, and bedside-accessible biomarkers. Among these, PhA has been extensively validated in other chronic conditions as a marker of cell membrane integrity and hydration status. Its prognostic utility in COPD was corroborated in multiple studies. For example, Maddocks et al. (2015) found that PhA outperformed FFMI in predicting short-term mortality following an exacerbation, highlighting its value as a functional biomarker [33]. De Blasio et al. (2019) reported that lower PhA and higher IR were associated with increased two-year mortality, even after adjusting for FEV1, inspiratory capacity, and 6 min walk distance [32].

While FFMI and FFM have historically been used to characterize sarcopenia, their prognostic utility was inconsistent in the studies included. Karanikas et al. (2021) and Choi et al. (2023) found significant associations in basic models, but these were lost when controlling for previous exacerbations [30,31]. This suggests that FFMI may reflect a broader disease phenotype but may not independently predict outcomes.

Importantly, this review also highlights a neglected intersection in COPD research: the coexistence of cardiovascular comorbidities, particularly heart failure. As shown by Faisy et al. (2000), an excluded study, ECW% estimated by BIA was significantly higher in patients with acute respiratory failure and associated with increased mortality [36]. Although this study was excluded due to its mixed patient population, its findings point toward the potential of BIA in identifying congestion, especially in differentiating between decompensated COPD and HFpEF.

Other excluded studies, such as Teixeira et al. (2021) [37] and Mjid et al. (2021) [38], underscored the impact of low muscle mass on functional limitation and hospital stay, further confirming that body composition is intricately linked to clinical prognosis. These studies, although not eligible for the primary analysis, enrich contextual understanding and support the physiological plausibility of BIA-based stratification.

### 4.2. Strengths and Limitations

The strengths of this review include a comprehensive and transparent search strategy, predefined inclusion criteria, and rigorous methodological appraisal using the Newcastle–Ottawa Scale. Studies included spanned various geographic regions and clinical settings, and most used objective, time-bound outcomes (mortality, hospitalizations, or exacerbations). The review focused on parameters that can be integrated into clinical practice with relative ease and low cost.

However, notable limitations exist. First, there was significant methodological heterogeneity across studies, particularly regarding BIA device types, electrode placement, frequency settings, and parameter definitions (e.g., cutoffs for PhA or FFMI). Second, adjustment for relevant confounders, particularly cardiovascular comorbidities, inflammatory status, and baseline physical activity, was inconsistent or absent. No study systematically explored the interplay between BIA-derived markers and cardiac function in COPD. Third, sample sizes were limited in several studies, and generalizability is limited due to gender-specific cohorts (e.g., Kobayashi et al. included only male patients). Lastly, observational design and retrospective elements in some studies (e.g., Rodrigues et al., Choi et al.) limit causal inference.

### 4.3. Implications for Practice

The findings of this review suggest that BIA-derived parameters, especially PhA, may serve as valuable tools for enhancing risk stratification in COPD. These markers are accessible, noninvasive, and repeatable over time, allowing clinicians to monitor changes during exacerbations, recovery, or therapeutic interventions. In particular, low PhA may help identify patients with compromised cellular integrity or early-stage sarcopenia.

This has tangible implications for personalized care. Patients with low PhA or FFMI may benefit from prioritized access to pulmonary rehabilitation, individualized nutritional support, and closer post-discharge follow-up. In settings with limited resources, BIA may also support triage decisions and highlight individuals at increased risk of clinical deterioration.

In addition, BIA may assist in detecting fluid overload, offering insight into possible cardiovascular comorbidity. While not a replacement for echocardiography or natriuretic peptide testing, parameters such as ECW% or impedance ratio may signal systemic congestion, prompting further cardiac evaluation. This could be particularly valuable in older adults with ambiguous dyspnea or overlapping symptoms of COPD and heart failure.

Beyond risk stratification, BIA may also have practical value in optimizing the timing of spirometric testing. In patients with fluid overload, spirometry performed during a congestive state may yield falsely obstructive results, leading to misdiagnosis of COPD and inappropriate therapeutic decisions. Identifying volume expansion through BIA could help defer or contextualize spirometry, ensuring it reflects true airway physiology rather than reversible hemodynamic effects. This application, though underexplored, holds potential to improve diagnostic accuracy and avoid over-treatment in patients with complex cardiopulmonary presentations.

To strengthen the role of BIA in COPD management, future studies should focus on:

- Standardizing methodology, including device calibration, patient posture, and frequency selection
- Defining validated cutoffs for PhA, FFMI, and ECW%, tailored to COPD populations
- Integrating comorbidities and biomarkers (e.g., NT-proBNP) into prognostic models
- Assessing longitudinal changes in BIA parameters following rehabilitation, nutrition, or diuretic therapy
- Incorporating BIA into composite risk scores and exploring advanced tools such as segmental BIA and machine learning-based phenotyping

These steps may help establish BIA as a core component of multidimensional COPD assessment.

## 5. Conclusions

BIA provides novel, noninvasive parameters, such as PhA, FFMI, and impedance ratio, that show promising prognostic utility in patients with COPD. This systematic review demonstrates that low PhA is consistently associated with an increased risk of exacerbations and mortality, while reductions in FFM may reflect broader functional and nutritional vulnerability.

Despite methodological heterogeneity and limited adjustment for comorbidities across studies, the findings support the integration of BIA into multidimensional risk assessment strategies. In addition to its prognostic value, BIA can support individualized clinical decision-making by identifying patients who may benefit from targeted rehabilitation, nutritional support, or intensified follow-up.

Moreover, its potential role in detecting systemic congestion could aid in distinguishing COPD from coexisting heart failure and in optimizing the timing of spirometry. By identifying volume overload, BIA may prevent false obstructive diagnoses and unnecessary treatments in patients with overlapping cardiopulmonary conditions.

Future well-designed, prospective, standardized, and longitudinal studies are essential to validate these applications and define the clinical thresholds necessary for integration into practice.

## Figures and Tables

**Table 1:** Summary of characteristics and findings of the included studies.

Author and Year	Study Design	Sample	Device	BIA Parameters	Conclusions
Kobayashi et al. 2024 [28]	Prospective observational cohort study	108, male	InBody S10	Segmental and whole-body PhA.	Segmental PhA in the lower limbs was associated with the incidence of severe COPD exacerbations in male patients and may serve as a noninvasive prognostic marker for severe COPD exacerbations.
Xie et al. 2024 [29]	Cross-sectional study with prognostic stratification via logistic regression.	159, male	InBody S10	BCM, BMC, ECW, ECW/ICW, ECW/TBW, FFM, FFMI, FM, FMI, ICW, Mineral, PBF, PhA, Protein, SLM, SMI, SMM, TBW, VFA.	Low PhA and/or high ECW/TBW were associated with frequent exacerbations. They may serve as noninvasive markers for exacerbation frequency in COPD.
Gomez-Martinez et al. 2023 [34]	Prospective cohort study	240, 51% male	BodyStat QuadScan 4000	ASMMI, ECW, FM, FFMI, IR, PhA, TBW.	PhA below the 50th percentile was independently associated with mortality. PhA may serve as independent prognostic marker of all-cause mortality in COPD.
Choi et al. 2023 [30]	Retrospective observational cohort study (multicenter)	253, 74.7% male	Not specified	ASMI, FMI, FFMI, SMI, TSMI.	Low muscle mass was associated with poor prognosis, particularly low TSMI was significantly associated with increased risk of acute exacerbation.
Karanikas et al. 2021 [31]	Prospective observational cohort study	76, 50% male	InBody S10	BFM, FFM, FFMI, PhA.	BIA parameters such as FFM and FFMI were associated with the incidence of AE in one year, but associations lost significance after adjusting for prior AE.
De Blasio et al. 2019 [32]	Prospective observational cohort study	210, 69% male	Human IM-Touch analyzer (DS Medica)	FM, FFM, FFMI, IR, PhA.	Higher IR and lower PhA were strong, independent predictors of 2-year all-cause mortality in COPD patients.
Rodrigues et al. 2018 [35]	Retrospective cohort	141, 56% male	Not specified	FFMI.	Cluster analysis using FFMI and other known mortality predictors outperformed BODE index in identifying 2-year mortality risk in COPD patients.
Maddocks et al. 2015 [33]	Prospective observational	502, 58.7% male	BodyStat QuadScan 4000	FFM, FFMI, PhA.	PhA was a marker of function and disease severity in stable COPD and a possible predictor for mortality.

Abbreviations: AE—Acute Exacerbation. ASMI (kg)—Appendicular Skeletal Muscle Mass. ASMMI (kg/m^2^)—Appendicular Skeletal Muscle Mass Index. BCM (kg)—Body Cell Mass. BFM (kg)—Body Fat Mass. BIA—Bioimpedance Analysis. BMC (kg)—Bone Mineral Content. BODE—Body mass index, airflow Obstruction, Dyspnea, and Exercise capacity index. COPD—Chronic Obstructive Pulmonary Disease. ECW (L or %)—Extracellular Water. ECW/ICW—Extracellular Water/Intracellular Water Ratio. ECW/TBW—Extracellular Water/Total Body Water Ratio. FFM (kg)—Fat-Free Mass. FFMI (kg/m^2^)—Fat-Free Mass Index. FM (kg)—Fat Mass. FMI (kg/m^2^)—Fat Mass Index. ICW (L)—Intracellular Water. IR—Impedance Ratio. Mineral (kg)—Mineral Mass. PBF (%)—Percent Body Fat. PhA (°)—Phase Angle. SLM (kg)—Soft Lean Mass. SMI (kg/m^2^)—Skeletal Muscle Index. SMM (kg)—Skeletal Muscle Mass. TBW (L or %)—Total Body Water. VFA (cm^2^)—Visceral Fat Area.

## Data Availability

No new data were created or analyzed in this study. Data sharing is not applicable to this article.

## Abbreviations

The following abbreviations are used in this manuscript:

AE	Acute Exacerbation
ASMI	Appendicular Skeletal Muscle Mass
ASMMI	Appendicular Skeletal Muscle Mass Index
BCM	Body Cell Mass
BFM	Body Fat Mass
BIA	Bioelectrical Impedance Analysis
BMC	Bone Mineral Content
BODE	Body mass index, airflow Obstruction, Dyspnea, and Exercise capacity index
CAR	CRP-to-albumin ratio
COPD	Chronic Obstructive Pulmonary Disease
CRP	C-reactive protein
DXA	Dual-energy X-ray Absorptiometry
ECW	Extracellular Water
ECW/ICW	Extracellular Water/Intracellular Water Ratio
ECW/TBW	Extracellular Water/Total Body Water Ratio
FEV_1_	Forced Expiratory Volume in One Second
FFM	Fat-Free Mass
FFMI	Fat-Free Mass Index
FM	Fat Mass
FMI	Fat Mass Index
GDF-15	Growth Differentiation Factor-15
HfpEF	Heart Failure with Preserved Ejection Fraction
HR	Hazard Ratio
ICW	Intracellular Water
IL-6	Interleukin-6
IL-8	Interleukin-8
IR	Impedance Ratio
MCR	Motoric Cognitive Risk
MeSH	Medical Subject Headings
MF-BIA	Multifrequency Bioelectrical Impedance Analysis
NLR	Neutrophil-to-lymphocyte Ratio
NOS	Newcastle-Ottawa Scale
NT-proBNP	N-terminal pro-B-type Natriuretic Peptide
OR	Odds Ratio
PBF	Percent Body Fat
PhA	Phase Angle
PRISMA	Preferred Reporting Items for Systematic Reviews and Meta-Analyses
RR	Relative Risk
SAA	Serum Amyloid A
SF-BIA	Single-Frequency Bioelectrical Impedance Analysis
SMI	Skeletal Muscle Index
SLM	Soft Lean Mass
SMM	Skeletal Muscle Mass
TBW	Total Body Water
TNF-α	Tumor Necrosis Factor-α
VFA	Visceral Fat Area

## Appendix A

### Appendix A.1. Search Strategies

Representative search strings used are as follows:

- Scopus:

TITLE-ABS-KEY (“bioimpedance” OR “bioelectrical impedance” OR “BIA” OR “multifrequency bioimpedance” OR “multi-frequency bioelectrical impedance”) AND (“COPD” OR “chronic obstructive pulmonary disease”) AND (“prognosis” OR “prognostic” OR “outcome” OR “mortality” OR “survival”).

- Web of Science:

TS = (“bioimpedance” OR “bioelectrical impedance” OR “BIA” OR “multifrequency bioimpedance” OR “multi-frequency bioelectrical impedance”) AND (“COPD” OR “chronic obstructive pulmonary disease”) AND (“prognosis” OR “prognostic” OR “outcome” OR “mortality” OR “survival”).

- PubMed:

(“Bioelectrical Impedance Analysis”[Mesh] OR bioimpedance[tiab] OR “bioelectrical impedance”[tiab] OR BIA[tiab] OR “multifrequency bioimpedance”[tiab] OR “multi-frequency bioelectrical impedance”[tiab]) AND (“Pulmonary Disease, Chronic Obstructive”[Mesh] OR COPD[tiab] OR “chronic obstructive pulmonary disease”[tiab]) AND (prognosis[tiab] OR prognostic[tiab] OR outcome[tiab] OR mortality[tiab] OR survival[tiab]).

### Appendix A.2. Data Extraction Forms

**Table A1:** Data extraction forms.

Author	Year	Country	Study Design	Sample Size	Follow-Up Duration	Baseline Characteristics	Device	BIA Parameters	Prognostic Outcome	Key Results	Conclusions
											

### Appendix A.3. Risk of Bias Assessment Tools

**Table A2:** Risk of bias assessment tools.

Author	Year	Study Design	Tool Used	Score	Level of Bias	Observations
Kobayashi et al. [28]	2024	Prospective cohort	Newcastle–Ottawa	7.5	Low	Clear follow-up and objective outcomes; limited by single-center, small all-male sample (external validity).
Xie et al. [29]	2024	Cross-sectional with logistic regression	Newcastle–Ottawa	5.5	Moderate	Retrospective outcome: logistic regression applied. Sample small and male-only.
Gomez-Martinez et al. [34]	2023	Prospective cohort	Newcastle–Ottawa	9	Low	Well-defined prospective cohort with standardized BIA, mortality outcome, and low bias risk.
Choi et al. [30]	2023	Retrospective cohort (multicenter)	Newcastle–Ottawa	7	Moderate	Multicenter retrospective cohort: outcome and follow-up clearly defined. Device variability not controlled.
Karanikas et al. [31]	2021	Prospective cohort	Newcastle–Ottawa	8	Low	Prospective study with multifrequency BIA and valid AE-COPD outcome; sample small but protocol clearly reported.
De Blasio et al. [32]	2019	Prospective cohort	Newcastle–Ottawa	8	Low	Prospective design with standardized BIA and adjusted analyses; single center and low event count are limitations.
Rodrigues et al. [35]	2018	Retrospective cohort	Newcastle–Ottawa	7	Moderate	Retrospective with validated mortality outcome; device not reported, and model lacks external validation.
Maddocks et al. [33]	2015	Prospective observational	Newcastle–Ottawa	8	Low	Large prospective cohort, standardized BIA; follow-up timing unclear, but outcome data robust.

### Appendix A.4. Summary of Findings from Included Studies

**Table A3:** Methodological characteristics of included studies.

Author	Year	Country	Study Design	Sample Size (n=)	Follow-Up Duration	Baseline Characteristics	Device
Kobayashi et al. [28]	2024	Japan	Prospective observational cohort study	108	3 years (median 1082 days)	Male patients with stable COPD. Mean values: age (years) 75.1 ± 7.9, BMI (Kg/m^2^) 22.7 ± 3.5, FEV_1_ (liters) 1.46 ± 0.58, FEV_1_% pred. 55.7 ± 20.7. GOLD stage (%): I 16.7, II 56.7, III 20, IV 6.7. Comorbidities: cardiovascular disease 20.4%.	InBody S10
Xie et al. [29]	2024	China	Cross-sectional study with prognostic stratification via logistic regression	159	1 year (retrospective)	Male patients (77 AE-COPD, 82 stable COPD). Mean values in AE-COPD: age (years) 67 ± 8.8, BMI (kg/m^2^) 18.6 ± 3.4, CAT 21. Mean values in stable COPD: age (years) 62.9 ± 8.3, BMI (kg/m^2^) 21.4 ± 4.1, CAT 13.5.	InBody S10
Gomez-Martinez et al. [34]	2023	Mexico	Prospective cohort study	240	7 years (mean 6.66 years)	51% male. Mean age (years) 72.3 ± 8.2, BMI (kg/m^2^) 28.4 ± 7.4, FEV1 (liters) 1.26 ± 0.63, FEV_1_ (%) 57.61 ± 23.55, GOLD stage (%) I and II 60.18, III and IV 39.82. Comorbidities: heart failure 42.08%.	BodyStat QuadScan 4000
Choi et al. [30]	2023	South Korea	Retrospective cohort (multicenter)	253	10 years (median 3.3 years)	74.7% male. Mean: age (years) 71.0, BMI (kg/m^2^) 22.8 (20.4–25.0). Comorbidities: heart failure 31.6%.	Not reported
Karanikas et al. [31]	2021	Greece	Prospective observational cohort study	76	12 months	50% male. Without AE (n = 25): FEV_1_ (%) 62.6 ± 20.9. With AE (n = 51): FEV_1_ (%) 48.5 ± 17.4.	InBody S10
De Blasio et al. [32]	2019	Italy	Prospective cohort study	210	24 months	69% male. Survivors: mean age 68.9 ± 8, BMI 26.2 ± 5.4, FEV_1_ (%) 41 ± 16, GOLD-IV (%) 29.3; Non-survivers: mean age 72.4 ± 8.3, BMI 23.8 ± 6.4, FEV_1_ (%) 36 ± 23.9, GOLD-IV (%) 51.7.	Human IM-Touch
Rodrigues et al. [35]	2018	Brazil	Retrospective cohort	141	24 months	56% male. Mean: age 70 ± 8, BMI (kg/m^2^) 24.6, FEV_1_ (%) 42 ± 17.	Not reported
Maddocks et al. [33]	2015	United Kingdom	Prospective observational	502	Follow-up: 90 days (mortality outcome); median cohort follow-up: 469 days	Stable COPD. 58.7% male. Median: age (years) 71, BMI (kg/m^2^) 24.7, FEV_1_ (%) 45.	BodyStat QuadScan 4000

Abbreviations: AE—Acute Exacerbation; BMI—Body Mass Index; CAT—The COPD Assessement Test; COPD—Chronic Obstructive Pulmonary Disease; FEV_1_—Forced Expiratory Volume in 1 s.

**Table A4:** Prognostic findings and conclusions.

Author (Year)	BIA Parameters	Prognostic Outcome	Key Results	Conclusions
Kobayashi et al. (2024) [28]	Segmental PhA (mean values): PhA-RA (4.96 ± 0.73), PhA-LA (4.92 ± 0.73), Ph-RL (4.61 ± 1.10), PhA-LL (4.44 ± 1.06), PhA-TR (5.04 ± 1.07). Whole-body PhA (4.83 ± 0.82).	Severe exacerbation of COPD (hospitalization/emergency department visit)	Low PhA for RL (HR = 3.50, 95% CI 1.33–9.20, *p*= 0.010), LL (HR = 3.26, 95% CI 1.18–9.04, *p* = 0.023) and LA (HR = 2.61, 95% CI 1.17–5.8, *p* = 0.020) were significant predictors for severe exacerbations (adjusted for age, FEV_1_% pred, CAT score, history of severe exacerbations in the past year). Whole-body PhA and trunk PhA were not significant. RL PhA had the best predictive performance (AUC = 0.73, 95% CI 0.64–0.82), followed by LL (AUC = 0.71, 95% CI 0.62–0.81). Cut-off values of PhA for predicting severe exacerbations: RL 4.5°, LL 4.5°, RA 5.0°.	Segmental PhA in the lower limbs may serve as a noninvasive prognostic marker for severe COPD exacerbations in male patients.
Xie et al. (2024) [29]	SMI (Kg/m^2^), FFMI (kg/m^2^), FMI (kg/m^2^), PBF (%), ECW/ICW, PhA (°).	Exacerbation phenotype (frequent vs. infrequent).	Lower PhA was associated with reduced odds of being a frequent exacerbator (OR = 0.396, C95% CI 0.164–0.957, *p* = 0.040), while higher ECW/TBW increased the odds (OR = 1.086, (5% CI 1.010–1.168, *p* = 0.026). Cut-off values: PhA < 4.85° (AUC = 0.753, 95% CI 0.624–0.882), ECW/TBW > 0.393 (AUC= 0.744, 95 %CI 0.611–0.876).	PhA and ECW/TBW may serve as noninvasive markers for exacerbation risk stratification in COPD.
Gomez-Martinez et al. (2023) [34]	Mean values: FFMI (kg/m^2^) 16.62 ± 3.42, IR 0.83, TBW (%) 51.64 ± 8.17, ECW (%) 27.77 ± 6.03, PhA 5.05 ± 0.96	All-cause mortality	PhA (per degree) was protective (HR = 0.59, 95% CI 0.37–0.94, *p* = 0.026). PhA < 50th percentile associated with increased mortality risk (HR = 3.47, 95% CI 1.45–8.29, *p* = 0.005). Sarcopenia (HR = 2.10, 95% CI 1.02–4.33, *p* = 0.043) was also associated with higher mortality. IR was not significant in multivariate analysis.	PhA and functional indicators of muscle mass may serve as independent prognostic markers of all-cause mortality in COPD.
Choi et al. (2023) [30]	FMI (kg/m^2^) 6.0 (4.5–8.0), FFMI (kg/m^2^) 16.4 ± 2.2, SMI (kg/m^2^) 14.2 (12.7–15.5).	AE-COPD, pneumonia development, outpatient/ER visits, (within 1 year). All-cause in hospital mortality.	Low TSMI—independently associated with acute exacerbations (HR = 0.200, 95% CI 0.048–0.838, *p* = 0.028).	Low muscle mass, particularly low TSMI, is associated with increased risk of exacerbation.
Karanikas et al. (2021) [31]	FFM (kg), FFMI (kg/m^2^), PhA, BFM (%).	COPD acute exacerbation	In multivariate logistic regression (Model 1, adjusted for age, sex, and smoking index): FFM (OR = 0.876, 95% CI: 0.790–0.971, *p* = 0.012), FFMI (OR = 0.671, 95% CI: 0.502–0.897, *p* = 0.007) were significantly associated with a lower risk of acute COPD exacerbation within one year.	Associations lost significance after adjusting for prior AE; not independent prognostic factors.
De Blasio et al. (2019) [32]	FFM (Survivors 46.8 ± 8.5, non-survivors 43.2 ± 9.2, *p* = 0.031), FFMI, PhA (Survivors 5.2 ± 1.2, non-survivors 4.3 ± 1.2, *p* < 0.001).	2-year all-cause mortality	Cox regression model 3: PhA (B = −0.63, HR = 0.53, 95% CI: 0.36–0.77, *p* < 0.001); 50/5 IR (B = 0.30, HR = 1.38, 95% CI: 1.14–1.66, *p* < 0.001); 100/5 IR (B = 0.26, HR = 1.29, 95% CI: 1.30–1.49, *p* < 0.001); 250/5 IR (B = 0.15, HR = 1.16, 95% CI: 1.03–1.30, *p* < 0.001); PhA < 4.1° and IR > 0.843 strongly predicted mortality across quintiles.	IR and PhA are strong, independent predictors of mortality in COPD patients.
Rodrigues et al. (2018) [35]	FFMI (kg/m^2^).	2-year all-cause mortality	Cluster I (worse status, including lower FFMI) had 5.17× higher risk of 2-year mortality (HR = 5.17; 95% CI: 1.7–15.7; *p* = 0.004). FFMI < 16.7 kg/m^2^ among cut-offs significantly associated with high mortality risk (AUC = 0.750).	Cluster analysis using FFMI and other known mortality predictors outperformed BODE index in identifying 2-year mortality risk in COPD patients.
Maddocks et al. (2015) [33]	PhA (median: 4.7), FFMI (kg/m^2^).	Mortality	Low PhA associated with higher mortality (8.2% vs. 3.6%, *p* = 0.02), worse functional scores (ISW, 5STS, 4MGS, QMVC), and higher ADO/iBODE. PhA outperformed FFMI in predicting mortality.	PhA is a stronger functional and prognostic marker than FFMI in stable COPD and may aid in identifying high-risk patients.

Abbreviations: 4MGS—4 m gait speed; 5STS—5 sit-tot-stand time; ADO—Age Dyspnea Obstruction; AE—Acute Exacerbation; BFM—Body Fat Mass; BMI—Body Mass Index; CAT—The COPD Assessement Test; COPD—Chronic Obstructive Pulmonary Disease; ECW—Extracellular Water; ER—Emergency Room; FEV_1_—Forced Expiratory Volume in 1 s; FMI—Fat Mass Index; FFM—Fat Free Mass; FFMI—Fat Free Mass Index; iBODE—Body Mass Index, Obstruction, Dyspnea, Exercise Capacity Index; ICW—Intracellular Water; IR—Impedance Ratio; ISW—Incremental Shuttle Walk; LA—Left Arm; LL—Left Leg; PBF—Percentage of Body Fat; PhA—Phase Angle; QMVC—Quadriceps Strenght; RA—Right Arm; RL—Right Leg; SMI—Skeletal Muscle Index; TBW—Total Body Water; TR—Trunck; TSMI—Truncal Skeletal Muscle Mass Index.

### Appendix A.5. Excluded Studies and Reasons for Exclusion

**Table A5:** Excluded studies and reasons for exclusion.

	**Author**	**Year**	Title	Reason
1	Abbatecola et al.	2014	Body composition markers in older persons with COPD	Single-frequency BIA device
2	Ahmadi et al.	2020	Fortified whey beverage for improving muscle mass in chronic obstructive pulmonary disease: A single-blind, randomized clinical trial	No prognostic outcome assessment using BIA
3	Alsharaway	2019	Pulmonary rehabilitation outcome in chronic obstructive pulmonary disease patients with a different body composition	No prognostic outcome assessment using BIA
4	Attaway et al.	2024	Muscle loss phenotype in COPD is associated with adverse outcomes in the UK Biobank	Single-frequency BIA device
5	Boesch et al.	2024	Tracking Real-World Physical Activity in Chronic Obstructive Pulmonary Disease Over One Year: Results from a Monocentric, Prospective, Observational Cohort Study	Single-frequency BIA device
6	Budweiser et al.	2008	Nutritional depletion and its relationship to respiratory impairment in patients with chronic respiratory failure due to COPD or restrictive thoracic diseases	Single-frequency BIA device
7	Byun et al.	2017	Sarcopenia correlates with systemic inflammation in COPD	No prognostic outcome assessment using BIA
8	Camargo et al.	2023	Impact of Obstructive Sleep Apnea on Cardiac Autonomic Control during the Respiratory Sinus Arrhythmia Maneuver in Patients with Chronic Obstructive Pulmonary Disease	No prognostic outcome assessment using BIA
9	Camillo et al.	2008	Heart Rate Variability and Disease Characteristics in Patients with COPD	Single-frequency BIA device
10	Caro De Miguel et al.	2009	Relación entre la masa libre de grasa, la masa muscular, la fuerza de contracción voluntaria máxima del cuádriceps y el test de la marcha de 6 minutos en pacientes con enfermedad pulmonar obstructiva crónica	Single-frequency BIA device
11	Choi et al.	2022	Peak oxygen uptake and respiratory muscle performance in patients with chronic obstructive pulmonary disease: Clinical findings and implications	No prognostic outcome assessment using BIA
12	Chua et al.	2019	Body composition of Filipino chronic obstructive pulmonary disease (COPD) patients in relation to their lung function, exercise capacity and quality of life	No prognostic outcome assessment using BIA
13	Creutzberg et al.	2003	Efficacy of nutritional supplementation therapy in depleted patients with chronic obstructive pulmonary disease	Single-frequency BIA device
14	De Benedetto et al.	2018	Supplementation with Qter^®^ and Creatine improves functional performance in COPD patients on long term oxygen therapy	Single-frequency BIA device
15	De Blasio et al.	2017	Raw BIA variables are predictors of muscle strength in patients with chronic obstructive pulmonary disease	Single-frequency BIA device
16	Del Ponte and Marinari	2007	Body composition abnormalities in patients with COPD: limitations and intervention	No prognostic outcome assessment using BIA
17	Dela Coleta et al.	2008	Predictors of first-year survival in patients with advanced COPD treated using long-term oxygen therapy	Single-frequency BIA device
18	Demircioglu et al.	2020	Frequency of sarcopenia and associated outcomes in patients with chronic obstructive pulmonary disease	Single-frequency BIA device
19	Dore et al.	2000	Composition corporelle des patients ayand une bronchopneumopathie chronique obstructive—Comparaison de l’impedancemetrie bioelectrique et de l’anthropometrie	No prognostic outcome assessment using BIA
20	Eisner et al.	2007	Body composition and functional limitation in COPD	Single-frequency BIA device
21	Faisy et al.	2000	Bioelectrical impedance analysis in estimating nutritional status and outcome of patients with chronic obstructive pulmonary disease and acute respiratory failure	No prognostic outcome assessment using BIA
22	Finamore et al.	2018	Validation of exhaled volatile organic compounds analysis using electronic nose as index of COPD severity	No prognostic outcome assessment using BIA
23	Franssen et al.	2004	Effects of whole-body exercise training on body composition and functional capacity in normal-weight patients with COPD	Single-frequency BIA device
24	Franssen et al.	2005	Limb muscle dysfunction in COPD: Effects of muscle wasting and exercise training	Single-frequency BIA device
25	Giron et al.	2009	Nutritional state during COPD exacerbation: Clinical and prognostic implications	Single-frequency BIA device
26	Gologanu et al.	2014	Body composition in patients with chronic obstructive pulmonary disease.	No prognostic outcome assessment using BIA
27	Gurgun	2013	Effects of nutritional supplementation combined with conventional pulmonary rehabilitation in muscle-wasted chronic obstructive pulmonary disease: A prospective, randomized and controlled study	No prognostic outcome assessment using BIA
28	Hallin et al.	2011	Relation between physical capacity, nutritional status and systemic inflammation in COPD	No prognostic outcome assessment using BIA
29	Halytska and Stupnytska	2023	Clinical features of asthma-COPD overlap syndrome with comorbid type 2 diabetes mellitus	Single-frequency BIA device
30	Hamada et al.	2024	Phase angle measured by bioelectrical impedance analysis in patients with chronic obstructive pulmonary disease: Associations with physical inactivity and frailty	No prognostic outcome assessment using BIA
31	Hitzl et al.	2010	Nutritional status in patients with chronic respiratory failure receiving home mechanical ventilation: Impact on survival	Single-frequency BIA device
32	Hopkinson et al.	2008	Vitamin D receptor genotypes influence quadriceps strength in chronic obstructive pulmonary disease	Single-frequency BIA device
33	Hopkinson et al.	2007	A prospective study of decline in fat free mass and skeletal muscle strength in chronic obstructive pulmonary disease	Single-frequency BIA device
34	Hsu et al.	2014	Mini-Nutritional Assessment (MNA) is Useful for Assessing the Nutritional Status of Patients with Chronic Obstructive Pulmonary Disease: A Cross-sectional Study	Single-frequency BIA device
35	Ibrayeva et al.	2024	Bioimpedansometry in patients with chronic lung diseases in the context of the pulmonary-cardio-renal continuum	No prognostic outcome assessment using BIA; cross-sectional correlations only (based on English abstract, no full-text translation available
36	Ingadottir et al.	2018	Two components of the new ESPEN diagnostic criteria for malnutrition are independent predictors of lung function in hospitalized patients with chronic obstructive pulmonary disease (COPD)	No prognostic outcome assessment using BIA
37	Joppa et al.	2016	Sarcopenic Obesity, Functional Outcomes, and Systemic Inflammation in Patients With Chronic Obstructive Pulmonary Disease	Single-frequency BIA device
38	Juarez Morales et al.	2013	Anemia en pacientes ingresados por una exacerbación de EPOC. Influencia sobre el pronóstico de la enfermedad	No prognostic outcome assessment using BIA
39	Jungblut et al.	2009	The effects of physical training on the body composition of patients with copd	Single-frequency BIA device
40	Kaymaz et al.	2015	Comparison of the effects of neuromuscular electrical stimulation and endurance training in patients with severe chronic obstructive pulmonary disease	No prognostic outcome assessment using BIA
41	Kilduff et al.	2003	Clinical relevance of inter-method differences in fat-free mass estimation in chronic obstructive pulmonary disease	Single-frequency BIA device
42	Kobayashi et al.	2024	Phase angle as an indicator of physical activity in patients with stable chronic obstructive pulmonary disease	No prognostic outcome assessment using BIA
43	Kurosaki et al.	2009	Extent of emphysema on HRCT affects loss of fat-free mass and fat mass in COPD	No prognostic outcome assessment using BIA
44	Kyle et al.	2006	Body composition in patients with chronic hypercapnic respiratory failure	Single-frequency BIA device
45	Lima et al.	2011	Potentially modifiable predictors of mortality in patients treated with long-term oxygen therapy	Single-frequency BIA device
46	Luo et al.	2016	Fat-free mass index for evaluating the nutritional status and disease severity in COPD	No prognostic outcome assessment using BIA
47	Machado et al.	2023	Differential Impact of Low Fat-Free Mass in People With COPD Based on BMI Classifications: Results From the COPD and Systemic Consequences-Comorbidities Network	No prognostic outcome assessment using BIA
48	Marco et al.	2019	Malnutrition according to ESPEN consensus predicts hospitalizations and long-term mortality in rehabilitation patients with stable chronic obstructive pulmonary disease	Single-frequency BIA device
49	Marinari et al.	2013	Effects of nutraceutical diet integration, with coenzyme Q10 (Q-Ter multicomposite) and creatine, on dyspnea, exercise tolerance, and quality of life in COPD patients with chronic respiratory failure	Single-frequency BIA device
50	Martinez-Luna et al.	2022	Association between body composition, sarcopenia and pulmonary function in chronic obstructive pulmonary disease	Single-frequency BIA device
51	Maynard-Paquette et al.	2020	Ultrasound evaluation of the quadriceps muscle contractile index in patients with stable chronic obstructive pulmonary disease: Relationships with clinical symptoms, disease severity and diaphragm contractility	Single-frequency BIA device
52	McDonald et al.	2017	Chest computed tomography-derived low fat-free mass index and mortality in COPD	Single-frequency BIA device
53	Mete et al.	2018	Prevalence of malnutrition in COPD and its relationship with the parameters related to disease severity	Single-frequency BIA device
54	Minas et al.	2011	The Association of Metabolic Syndrome with Adipose Tissue Hormones and Insulin Resistance in Patients with COPD without Co-morbidities	Single-frequency BIA device
55	Mijd et al.	2021	Composition corporelle des patients atteints de bpco et son impact sur la fonction respiratoire	Single-frequency BIA device
56	Mogelberg et al.	2022	High-protein diet during pulmonary rehabilitation in patients with chronic obstructive pulmonary disease	Single-frequency BIA device
57	Monteiro et al.	2012	Obesity and Physical Activity in the Daily Life of Patients with COPD	Single-frequency BIA device
58	Mostert et al.	2000	Tissue depletion and health related quality of life in patients with chronic obstructive pulmonary disease	Single-frequency BIA device
59	Muller et al.	2006	Assessment of body composition of patients with COPD	No prognostic outcome assessment using BIA
60	Mursic et al.	2024	Body composition, pulmonary function tests, exercise capacity, and quality of life in chronic obstructive pulmonary disease patients with obesity	No prognostic outcome assessment using BIA
61	Naranjo-Hernandez et al.	2024	Smart Bioimpedance Device for the Assessment of Peripheral Muscles in Patients with COPD	No prognostic outcome assessment using BIA
62	Nezu et al.	2001	The effect of nutritional status on morbidity in COPD patients undergoing bilateral lung reduction surgery	Single-frequency BIA device
63	Norden et al.	2015	Nutrition impact symptoms and body composition in patients with COPD	No prognostic outcome assessment using BIA
64	Ogasawara et al.	2018	Effect of eicosapentaenoic acid on prevention of lean body mass depletion in patients with exacerbation of chronic obstructive pulmonary disease: A prospective randomized controlled trial	No prognostic outcome assessment using BIA
65	Orea-Tejeda et al.	2022	The impact of hydration status and fluid distribution on pulmonary function in COPD patients	No prognostic outcome assessment using BIA
66	Ovsyannikov et al.	2019	Cardiometabolic risk factors in patients with chronic obstructive pulmonary disease and obesity	Single-frequency BIA device
67	Persson et al.	2015	Vitamin D, vitamin D binding protein, and longitudinal outcomes in COPD	Single-frequency BIA device
68	Pison et al.	2004	Effects of home pulmonary rehabilitation in patients with chronic respiratory failure and nutritional depletion	Single-frequency BIA device
69	Ramires et al.	2012	Resting energy expenditure and carbohydrate oxidation are higher in elderly patients with COPD: A case–control study	Single-frequency BIA device
70	Ramirez-Fuentes et al.	2019	Ultrasound assessment of rectus femoris muscle in rehabilitation patients with chronic obstructive pulmonary disease screened for sarcopenia: correlation of muscle size with quadriceps strength and fat-free mass	Single-frequency BIA device
71	Rodrigues et al.	2020	Are the Effects of High-Intensity Exercise Training Different in Patients with COPD Versus COPD + Asthma Overlap?	No prognostic outcome assessment using BIA
72	Rubinsztajn et al.	2015	Correlation between hyperinflation defined as an elevated RV/TLC ratio and body composition and cytokine profile in patients with chronic obstructive pulmonary disease	Single-frequency BIA device
73	Rubinsztajn et al.	2016	Body composition analysis performed by bioimpedance in patients with chronic obstructive pulmonary disease	Single-frequency BIA device
74	Sabino et al.	2010	Nutritional status is related to fat-free mass, exercise capacity and inspiratory strength in severe chronic obstructive pulmonary disease patients	Single-frequency BIA device
75	Saglam et al.	2013	Relationship between obesity and respiratory muscle strength, functional capacity, and physical activity level in patients with chronic obstructive pulmonary disease	Single-frequency BIA device
76	Sanchez et al.	2011	Anthropometric midarm measurements can detect systemic fat-free mass depletion in patients with chronic obstructive pulmonary disease	Single-frequency BIA device
77	Scarlata et al.	2021	Association between frailty index, lung function, and major clinical determinants in chronic obstructive pulmonary disease	No prognostic outcome assessment using BIA
78	Schols et al.	2005	Body composition and mortality in chronic obstructive pulmonary disease	Single-frequency BIA device
79	Seymour et al.	2009	Ultrasound measurement of rectus femoris cross-sectional area and the relationship with quadriceps strength in COPD	Single-frequency BIA device
80	Sepulveda Loyola et al.	2024	Impact of pre-sarcopenia and sarcopenia on biological and functional outcomes in individuals with chronic obstructive pulmonary disease: a cross-sectional study	No prognostic outcome assessment using BIA
81	Slinde et al.	2005	Body composition by bioelectrical impedance predicts mortality in chronic obstructive pulmonary disease patients	Single-frequency BIA device
82	Souza et al.	2019	Inspiratory muscle strength, diaphragmatic mobility, and body composition in chronic obstructive pulmonary disease	No prognostic outcome assessment using BIA
83	Texeira et al.	2021	Low Muscle Mass Is a Predictor of Malnutrition and Prolonged Hospital Stay in Patients With Acute Exacerbation of Chronic Obstructive Pulmonary Disease: A Longitudinal Study	Single-frequency BIA device
84	Thibault et al.	2010	Assessment of nutritional status and body composition in patients with COPD: Comparison of several methods	Single-frequency BIA device
85	Tomita et al.	2024	Impact of Energy Malnutrition on Exacerbation Hospitalization in Patients with Chronic Obstructive Pulmonary Disease: Retrospective Observational Study	No prognostic outcome assessment using BIA
86	Vakhlamov and Tyurikova	2015	Rationale for the use of new methods of investigation of metabolic syndrome in the diagnosis and treatment of patients with broncho-obstructive diseases	Single-frequency BIA device
87	Vestbo et al.	2006	Body mass, fat-free body mass, and prognosis in patients with chronic obstructive pulmonary disease from a random population sample: Findings from the Copenhagen City Heart Study	Single-frequency BIA device
88	Waatevik et al.	2012	Different COPD characteristics are related to different outcomes in the 6 min walk test	Single-frequency BIA device
89	Waatevik et al.	2016	Oxygen desaturation in 6 min walk test is a risk factor for adverse outcomes in COPD	Single-frequency BIA device
90	Welch et al.	2025	Establishing Predictors of Acute Sarcopenia: A Proof-OfConcept Study Utilising Network Analysis	Mixed population; no prognostic analysis in COPD subgroup
91	Yogesh et al.	2024	The triad of physiological challenges: investigating the intersection of sarcopenia, malnutrition, and malnutrition-sarcopenia syndrome in patients with COPD—a cross-sectional study	No prognostic outcome assessment using BIA
92	Yoshikawa and Kanazawa	2021	Association of plasma adiponectin levels with cellular hydration state measured using bioelectrical impedance analysis in patients with COPD	No prognostic outcome assessment using BIA
93	Yoshimura et al.	2018	Interdependence of physical inactivity, loss of muscle mass and low dietary intake: Extrapulmonary manifestations in older chronic obstructive pulmonary disease patients	No prognostic outcome assessment using BIA
